# Cannabidiol (CBD) Inhibits *Streptococcus oralis* Growth and Biofilm Formation, While Maintaining Human Gingival Epithelial Cell Viability: An In Vitro Study

**DOI:** 10.1155/ijod/4609857

**Published:** 2026-08-12

**Authors:** Celina Cruz Mainardes, Omayma Amri, Denise M. Palomari Spolidorio, Mahmoud Rouabhia

**Affiliations:** ^1^ Oral Ecology Research Group (GREB), Dental Faculty, Laval University, 2420 rue de la Terrasse, Quebec QC G1V 0A6, Canada, ulaval.ca; ^2^ Department of Physiology and Pathology, School of Dentistry, São Paulo State University (UNESP), Araraquara 14801–903, São Paulo, Brazil, unesp.br

**Keywords:** biofilms, cannabidiol (CBD), epithelial cells, oral health, *S. oralis*

## Abstract

**Background:**

The oral ecosystem harbors multiple microorganisms, including *Streptococcus oralis* (*S. oralis*), which contributes to biofilm formation and microbial virulence. To eliminate oral biofilms, mechanical intervention is combined with antimicrobial agents such as chlorhexidine, but these have limited effects. Such intervention could benefit natural antimicrobial compounds, including cannabidiol (CBD).

**Aim:**

This study aims to evaluate the effect of CBD on reducing *S. oralis* growth and decreasing its biofilm‐forming capacity, as well as its interaction with human gingival epithelial cells, to explore its potential application as an oral antimicrobial agent.

**Methodology:**

*S. oralis* was cultured in the presence of different concentrations of CBD. Bacterial growth was evaluated at different time points postexposure to CBD. Bacterial biofilm formation was investigated after 3 days of exposure to CBD using histological and quantitative analyses. The interaction between CBD and gingival epithelial cells was assessed using cell morphology, cell adhesion, and cell viability/proliferation assays.

**Results:**

CBD inhibited planktonic growth of *S. oralis* in a concentration‐dependent manner with a minimum inhibitory concentration (MIC) of 6.25 μg/mL and a minimum bactericidal concentration (MBC) of 25 μg/mL. CBD also significantly (*p*  < 0.01) decreased *S. oralis* biofilm by disrupting its architecture. The effect on the bacterial growth and its capacity to form biofilms was observed even with a low concentration (3.12 μg/mL) of CBD. Given that antimicrobial molecules should be effective against biofilm‐associated bacteria while maintaining compatibility with host tissues, this study showed that concentrations (3.12, 6.25, and 12.5 μg/mL) of CBD we tested have anti‐*S. oralis* effect maintained human gingival epithelial cell viability.

**Conclusion:**

CBD exhibited a significant antimicrobial effect against *S. oralis*. Although the bactericidal concentration was higher than the range tested in gingival epithelial cells, low and intermediate CBD concentrations inhibited *S. oralis* growth and biofilm formation while maintaining cell viability after 24 h of exposure. These findings provide preliminary evidence supporting further investigation of the antimicrobial and antibiofilm properties of CBD in oral health contexts.

## 1. Introduction

Biofilms are highly organized microbial communities in which microorganisms adhere to surfaces and are embedded within an extracellular polymeric substance (EPS) matrix, contributing to microbial protection [1–4]. The most accessible biofilm in the human body is the dental plaque in the oral cavity [5, 6]. Oral biofilms can be associated with various oral diseases, including dental caries, periodontitis, and candidiasis [1, 6]. The oral biofilm’s structural and functional resilience represents a significant clinical challenge as the EPS matrix provides microbial protection against antibacterial and antifungal molecules, limiting their penetration to reach the microorganisms embedded within it. This EPS matrix blocks the host immune defense mechanisms, allowing microorganisms to form biofilms [2, 3].

The oral ecosystem contains multiple microorganisms that cohabit in the mouth. Among these microorganisms are *Streptococcus* spp., a group of Gram‐positive bacteria known to predominate in the early stages of oral biofilm formation and to be essential for microbial succession [5]. Among them, *Streptococcus oralis* (*S. oralis*) is identified as a pioneer colonizer, contributing to biofilm stability [6, 7] and facilitating the adhesion of other species, including *Actinomyces*, *Veillonella*, *Candida albicans*, and *Porphyromonas gingivalis*, promoting multispecies biofilm formation and microbial virulence [8, 9]. Through the oral biofilms, *S. oralis* may enter the bloodstream and could cause subacute infective endocarditis [8].

Clinical strategies to eliminate biofilm rely on mechanical removal combined with antimicrobial agents such as chlorhexidine [10, 11]. Chlorhexidine and other antimicrobial agents are efficient at controlling microbial growth but are associated with important limitations, including tooth staining and disruption of the oral microbiota, which limit their long‐term preventive use [10, 11]. These limitations have stimulated the search for alternative approaches to better control biofilm formation without compromising oral homeostasis.

In this context, cannabidiol (CBD) has been increasingly investigated as a bioactive compound with antimicrobial and host‐modulatory properties [12–14]. In dentistry, CBD has been suggested for its activity against *Streptococcus mutans* and *P. gingivalis* [13, 14]. However, the available evidence remains concentrated mainly on pathogens such as *S. mutans*, *Enterococcus faecalis*, *Candida albicans*, and periodontal‐associated species. In contrast, the effect of CBD on early oral colonizers remains insufficiently explored [15].

To be used clinically, a plant‐derived antimicrobial product, such as *Cannabis sativa*, should be compatible with human cells and tissues and should not cause tissue irritation. In this regard, when used in the oral cavity, the first contact of CBD will be with the gingival epithelial cells. These cells are essential components of the periodontal tissues and contribute to maintaining mucosal integrity. Beyond serving as a physical barrier between the oral cavity and the external environment, gingival epithelial cells actively participate in tissue repair and modulate local immune responses [16].

Cell adhesion is a key process in cellular communication and regulation of cell behavior, being indispensable for tissue development, organization, and maintenance [16, 17]. Therefore, when proposing a new compound for oral application, it is important to evaluate parameters such as cell adhesion and proliferation as these processes are directly related to tissue integrity and the preservation of oral homeostasis.

Despite the growing interest in the use of CBD in dentistry, its antimicrobial effects on pioneer streptococci involved in the early stages of oral biofilm formation, such as *S. oralis*, remain largely unexplored. We hypothesized that CBD would exhibit concentration‐dependent antimicrobial and antibiofilm activities against *S. oralis* while maintaining the viability of the host cells (gingival epithelial cells). Accordingly, the present study aimed to (i) evaluate the effect of CBD against *S. oralis* growth and biofilm formation and (ii) evaluate the effect of CBD on human gingival epithelial cell adhesion and viability/proliferation.

## 2. Material and Methods

### 2.1. Study Design and Ethical Statement

**Figure 1:**
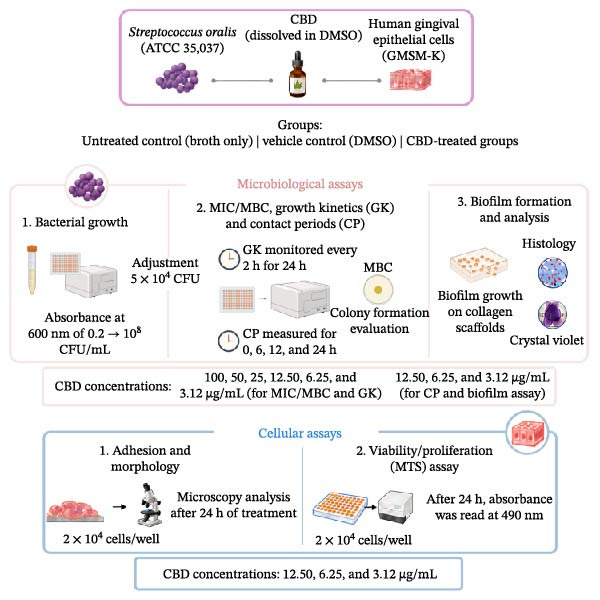
Graphical abstract of the experimental methodology. Schematic representation of the experimental workflow used to evaluate the antimicrobial and antibiofilm effects of cannabidiol (CBD) against *S. oralis* ATCC 35037 and its compatibility with human gingival epithelial cells (GMSM‐K). CBD was dissolved in dimethyl sulfoxide (DMSO), and the experimental groups included untreated control, vehicle control, and CBD‐treated groups. Microbiological assays included bacterial growth evaluation, MIC/MBC determination, growth kinetics, contact‐period analysis, colony formation assessment, and biofilm formation on collagen scaffolds followed by histological and crystal violet analyses. Cellular assays included evaluation of gingival epithelial cell adhesion, morphology, and viability/proliferation after CBD exposure. Different CBD concentrations were tested according to each assay, as indicated in the scheme.

This study was conducted as an in vitro experimental investigation (Figure 1). It did not involve human participants, animals, clinical data, identifiable human tissues, or clinical samples. The experiments were performed using a bacterial reference strain and commercially available human gingival epithelial cells. Therefore, ethical approval was not required. The study was reported according to the CRIS guidelines for in vitro studies.

The experimental design included evaluating CBD against *S. oralis* planktonic growth and biofilm formation as well as assessing its effects on human gingival epithelial cell viability and proliferation. The experimental groups included untreated control, vehicle control, and CBD‐treated groups at different concentrations. The main analyses included bacterial growth inhibition, biofilm formation assessment, histological evaluation, and cell viability analysis. A schematic representation of the study design is presented in Figure 1.

### 2.2. Outcomes

The primary outcome of this study was the antimicrobial effect of CBD against *S. oralis*, assessed by measuring planktonic bacterial growth inhibition and determining the minimum inhibitory concentration (MIC) after 24 h of exposure. Bacterial growth was monitored kinetically over 24 h by measuring the optical density (OD) at 600 nm and was additionally assessed at predefined time points.

The secondary outcomes included an evaluation of bactericidal activity, assessed by determining the minimum bactericidal concentration (MBC) after 24 h of CBD exposure, followed by subculture onto brain–heart infusion (BHI) agar. We also evaluated the antibiofilm activity after 3 days of CBD exposure using crystal violet (CV) staining for biofilm biomass quantification and histological analysis for biofilm architecture. Finally, the effect of CBD on human gingival epithelial cell viability and proliferation was assessed after 24 h using cell morphology and adhesion assays and an MTS‐based cell viability/proliferation assay.

### 2.3. CBD

The CBD used in this study was a pure (purity ≥ 98%) CBD extracted from an isolate of *Cannabis sativa* [18]. The CBD powder was dissolved in dimethyl sulfoxide (DMSO) to form a 5 mg/mL stock solution. From this stock, serial dilutions were performed to obtain the desired treatment concentrations. In all assays, a vehicle referring to the same DMSO level as in the highest CBD concentration was included to assess possible solvent effects on bacterial growth.

### 2.4. Bacterial Strain and Growth Conditions

Growth conditions for *S. oralis* in this study were adapted from previously published methods for *S. mutans* [19]. The *S. oralis* strains used in this study (ATCC 35,037) were reactivated and grown aerobically at 37°C in a BHI medium supplemented with 0.1% glucose. *S. oralis* cultures were incubated overnight, collected, and subsequently adjusted to a concentration of 10^7^ CFU/mL based on OD measured at 600 nm using a xMark microplate spectrophotometer (Bio‐Rad, Mississauga, ON, Canada). To convert absorbance to CFU, we used the following formula: Absorbance at 600 nm of 0.2 corresponds to 10^8^ CFU/mL.

### 2.5. To Evaluate the Effect of CBD on *S. oralis* Growth Over Time

*S. oralis* (5 × 10^4^ CFU) was seeded in triplicate in the wells of a 96‐well plate in 200 µL of BHI in the presence of CBD at concentrations ranging from 100 to 3.12 µg/mL. The vehicle control consisted of *S. oralis* cultured in BHI containing the highest volume of DMSO, at the highest final concentration in the CBD‐treated groups. Eight wells per condition were included. The plates were incubated at 37°C in a Synergy microplate reader (BioTek, Winooski, VT, USA), and growth was monitored every 2 h for 24 h by measuring the absorbance at 600 nm. Four biological replicates were made (*n* = 4).

### 2.6. To Evaluate the Growth of *S. oralis* at Different Contact Periods With CBD

*S. oralis* (5 × 10^4^ CFU) was seeded in triplicate in the wells of a 96‐well plate in 200 µL of BHI in the presence of CBD at concentrations ranging from 100 to 3.12 µg/mL. Vehicle control consisted of *S. oralis* being cultured in BHI containing the highest volume of DMSO as the highest final concentration of CBD in the CBD‐treated groups. Plates were incubated for 0, 6, 12, and 24 h at 37°C in a humid atmosphere with 5% CO_2_. After each incubation period, absorbance was measured at 600 nm using the xMark microplate spectrophotometer (Bio‐Rad, Mississauga, ON, Canada). Results are presented as the mean ± standard deviation (SD) of four biological replicates (*n* = 4). These experiments allowed the determination of the MIC of CBD. The MIC was defined as the lowest CBD concentration capable of inhibiting detectable bacterial growth after 24 h, as determined by OD measurements [20].

### 2.7. Identification of the MBC of CBD

*S. oralis* (5 × 10^4^ CFU) was seeded in triplicate in the wells of a 96‐well plate in 200 µL of BHI in the presence of CBD at concentrations ranging from 100 to 3.12 µg/mL. Plates were incubated for 24 h at 37°C in a humid atmosphere with 5% CO_2_. After this incubation period, 5 µL aliquots from each cell suspension of *S. oralis*, exposed or not to CBD for 24 h, were placed as a drop on the surface of BHI agar and incubated for an additional 24 h under the same growth conditions described above. Macroscopic observations were made to evaluate the absence of bacterial growth (no colony formation). The plates were then scanned and presented, showing the lowest CBD concentration capable of eliminating visible bacterial growth, as indicated by the CBD‐MBC [20]. Four biological replicates were performed (*n* = 4).

### 2.8. Effect of CBD on *S. oralis* Biofilm Formation

Biofilms of *S. oralis* were obtained using porous collagen scaffolds (Collatape, Zimmer Dental Inc., Carlsbad, CA, USA), which promote bacterial penetration and adhesion due to the bacterium’s affinity for collagen [19]. Use of these scaffolds also enables biofilm manipulation with minimal cell loss, thereby preserving its three‐dimensional structure. Biofilm formation was performed using 5 mm × 5 mm samples of the porous scaffolds placed in the wells of a 24‐well plate. The three‐dimensional porous collagen membranes (scaffold) were inoculated with 50 µL of *S. oralis* suspension (5 × 10^4^ CFU) in triplicate. The plates were incubated for 1 h at 37°C, allowing for initial bacterial adhesion to the scaffold. At this time, *S. oralis*‐seeded scaffolds were exposed to one of the following CBD concentrations (12.50, 6.25, or 3.12 µg) in 150 µL of BHI medium in triplicate. After 1 h of incubation, to ensure penetration of the lipophilic compound into the collagen membrane and into the adherent bacteria, 950 µL of fresh BHI medium was added to each well, with CBD used at the following concentrations (12.50, 6.25, and 3.12 µg/mL). Negative controls (biofilms without CBD; only DMSO) and positive controls (biofilms treated with 5 µg/mL penicillin/streptomycin) were included in the experiments. Cultures were maintained at 37°C for 3 days, with renewal of the medium and CBD treatment every 24 h. Three biological replicates were performed.

At the end of the third day, collagen membranes were washed three times with phosphate‐buffered saline solution (PBS) and fixed with 4% paraformaldehyde for 24 h, as previously reported [21]. Samples were then subjected to qualitative (histological) and quantitative (CV staining) analyses.

### 2.9. Histological Analysis

Histological processing and Masson’s trichrome staining were performed following standard protocols described in the literature [22–24]. Briefly, after fixation in paraformaldehyde, biofilms were washed with PBS, dehydrated through a graded ethanol series, cleared in xylene, and embedded in paraffin. Thin sections (5–7 µm) were cut with a microtome and mounted on glass slides. Sections were rehydrated, post‐fixed in Bouin solution, and stained using Weigert’s iron hematoxylin, Biebrich scarlet/acid fuchsin, a phosphotungstic/phosphomolybdic acid solution, and aniline blue, followed by differentiation with 1% acetic acid and graded alcohol dehydration [23]. After final dehydration and clearing, slides were mounted and analyzed with a high‐resolution digital microscope (Echo Laboratories– Bio‐Rad, San Diego, CA, USA). The system is equipped with high‐resolution optics and an integrated digital camera, both controlled by the proprietary Revolve 4.0 software (Echo Laboratories). Using the software, real‐time adjustments of focus, contrast, brightness, and magnification were performed to ensure the image quality. For each biofilm condition, four glass slides were prepared, each containing two sections. After staining, 10 images were captured per condition, covering distinct and representative areas. Images demonstrating structural definition and histological representativeness were selected for biofilm structural analysis. To minimize assessment bias, histological images were coded before analysis, and biofilm structural evaluation was performed by an investigator blinded to the experimental groups. Group allocation was revealed only after image selection and qualitative assessment were completed.

### 2.10. Evaluate Biofilm Formation Using the CV Assay

Biofilm biomass was quantified using the CV assay, which stains the biofilm (live cells and the extracellular matrix they secrete), as previously reported [21]. Briefly, biofilm samples were transferred to a sterile six‐well plate and left to dry for 24 h under a chemical flow hood. They were then layered with 500 µL of 0.1% CV solution and incubated for 15 min at room temperature. *S. oralis*‐free collagen scaffolds were included as a control. After CV staining, the collagen membranes were thoroughly washed with distilled water until the unbound dye was completely removed and then left to dry for 24 h under a chemical hood. Subsequently, 2.5 mL of 30% acetic acid was added to each well, and the samples were gently agitated to dissolve the bound CV dye. The obtained solutions were transferred (200 µL/well) to the wells of a 96‐well plate in quadruplicate, and the absorbance was measured at 595 nm using the xMark microplate spectrophotometer.

### 2.11. Evaluate the Interaction of CBD With Human Gingival Epithelial Cells

#### 2.11.1. Cells and Cell Culture Conditions

The gingival epithelial cells used in this study correspond to the non‐cancerous human gingival epithelial cell line GMSM‐K (CVCL_6A82) [25]. Gingival epithelial cells were cultured in Dulbecco’s Modified Eagle Medium/Ham’s F‐12 (DMEM), supplemented with 10% fetal bovine serum (FBS). The culture medium was renewed three times per week. When cultures reached ~80% confluence, cells were detached using 0.25% trypsin, washed, and resuspended in DMEM supplemented with FBS [26, 27] and used for our experiments.

#### 2.11.2. Effects of CBD on Gingival Epithelial Cell Adhesion and Cell Morphology

Gingival epithelial cells (2 × 10^5^ cells/well in 1 mL), in triplicate, were seeded in 6‐well tissue culture plates and immediately exposed to CBD at one of the following concentrations (3.12, 6.25, and 12.50 µg/mL), along with a vehicle control (DMSO) and a negative control referring to cells cultured in CBD and DMSO‐free medium. The highest concentration of DMSO in the treated samples was 0.25% (v/v), which, in our studies, was shown to be noncytotoxic to the cells. Cells were cultured in the presence or absence of CBD for 24 h at 37°C in a humidified atmosphere containing 5% CO_2_. After the incubation period, adherent and nonadherent cells in each well were examined using an inverted light microscope (10x objective) (Nikon Canada, Mississauga, ON, Canada). Photos from experimental conditions (control and CBD‐treated cells) were captured using a Coolpix 950 camera (Nikon Canada).

Cell morphology was evaluated after CV staining of adherent cells in each culture condition (CBD‐exposed and non‐exposed). Briefly, wells were washed with PBS, and then cells were fixed with 4% paraformaldehyde overnight, washed with PBS, and stained with CV, as described above [21–23]. The adherent CV‐stained cells were photographed. Four biological replicates were performed (*n* = 4).

#### 2.11.3. Effects of CBD on Cell Viability and Proliferation

Gingival epithelial cells were seeded at a density of 2 × 10^4^ cells/well in 6‐well plates and incubated for 24 h in a humidified atmosphere containing 5% CO_2_ at 37°C. After this culture period, the medium was refreshed, and adherent cells were exposed or not to CBD at 3.12, 6.25, and 12.50 µg/mL. As controls, cells were cultured in the presence of 0.25% DMSO (v/v) or in the culture medium. All culture conditions (cells exposed or not to CBD) were incubated in a humidified atmosphere containing 5 % CO_2_ at 37°C for 24 h. After this period, the medium was refreshed, and cell viability/proliferation was assessed using an MTS‐colorimetric assay (ab197010, Abcam Inc., Toronto, ON, Canada), as previously reported [27]. Briefly, a volume of 100 µL/mL of CellTiter‐96 Aqueous One Solution reagent was added to each well, and cultures were incubated for 3 h at 37°C in 5% CO_2_. At the end of the incubation period, 150 µL of the reaction mixture was transferred to a 96‐well plate, and the absorbance was measured at 490 nm using a spectrophotometer. Five biological replicates were performed (*n* = 5); results were expressed as mean (SD).

### 2.12. Statistical Analyses

The number of biological replicates was based on previous in vitro studies using similar microbiological and cell viability assays and on experimental feasibility [18, 19, 21]. No formal a priori sample size calculation was performed. Random allocation and allocation concealment were not applicable because this was an in vitro assay using predefined experimental conditions. When possible, samples and images were analyzed by an investigator independent of the experimental procedures.

The statistical analyses were applied to outcomes related to planktonic growth inhibition (bacteriostatic and bactericidal activities), biofilm biomass, and cell viability. Prior to ANOVA, data distribution and homogeneity of variance were assessed using the Shapiro–Wilk test and the Brown–Forsythe test, respectively. MIC, antibiofilm assays, and cell viability were analyzed by one‐way ANOVA, followed by Dunnett’s multiple comparisons test versus DMSO. The growth kinetics assay was analyzed by two‐way repeated‐measures ANOVA (factors: CBD concentration and time), followed by post hoc multiple comparisons using Dunnett’s test to compare each CBD‐treated condition with the control and, when applicable, Tukey’s test for pairwise comparisons among CBD concentrations. The data were recorded as the mean ± SD. Statistical significance was set at *p*  < 0.05. All analyses were performed in GraphPad Prism version 10 (GraphPad Software, San Diego, CA, USA).

## 3. Results

### 3.1. Long‐Term Exposure to CBD Reduced *S. oralis* Growth

**Figure 2:**
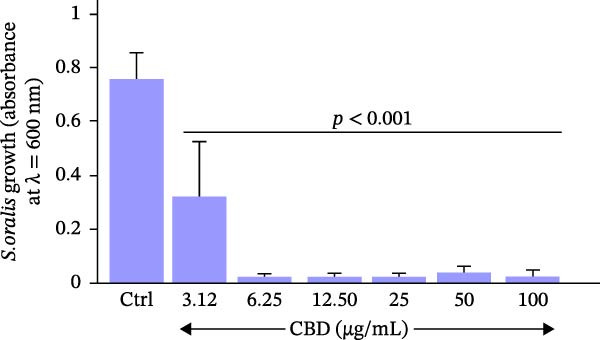
CBD decreased the *S. oralis* growth after long‐term contact. The bacterial cells were cultured in the presence of different concentrations of CBD for 24 h, and then bacterial growth was evaluated by absorbance at 600 nm. Four biological replicates were performed (*n* = 4) and means ± SD were presented. DMSO was included as a control. Statistical analysis revealed significant effects (*p*  < 0.001) when comparing the CBD effect to the control (DMSO).

Exposure of *S. oralis* to CBD for 24 h resulted in a significant decrease in bacterial growth (Figure 2). Indeed, the vehicle (DMSO) used to dissolve CBD did not affect bacterial growth (absorbance = 0.75 ± 0.10). However, all tested CBD concentrations showed growth inhibition compared to the DMSO control ( ^∗∗∗^ *p*  < 0.0001). The CBD decreased *S. oralis* growth, even at 3.12 µg/mL, as the absorbance was 0.32 ± 0.27 after 24 h of incubation, compared to that of DMSO (0.75 ± 0.10). However, despite this statistically significant reduction, 3.12 µg/mL did not meet the MIC criterion as detectable bacterial growth persisted after 24 h. Therefore, 6.25 µg/mL, which showed an absorbance of 0.02 ± 0.02, was determined as the MIC. Given this interesting inhibitory effect of CBD on *S. oralis* growth at 24 h of contact, we evaluate whether this effect occurs at earlier time points.

### 3.2. Kinetic Inhibition of *S. oralis* Growth by CBD

**Figure 3:**
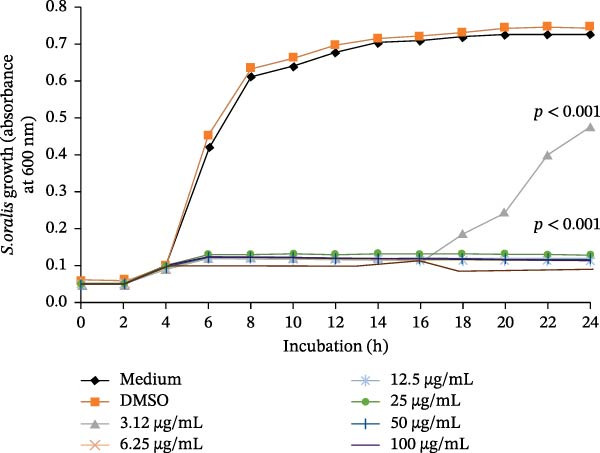
Kinetics of the inhibitory effect of CBD on bacterial growth over 24 h. Growth curves showing absorbance at 600 nm over different exposure times to CBD at different concentrations. The growth of the CBD‐exposed bacteria was compared to that of the vehicle DMSO. Data points represent means of different biological replicates (*n* = 4). Statistical analysis revealed significant effects (*p*  < 0.001) when comparing the CBD effect to the control (DMSO).

The bacteria were cultured in the absence or presence of CBD at different concentrations, with a follow‐up every 2 h. It was observed (Figure 3) that all tested CBD concentrations maintained the *S. oralis* growth at its lowest rate up to 16 h. At 18 h post contact, only the lowest concentration (3.12 μg/mL) showed growth of *S. oralis*, which continued increasing with subsequent culture periods (at 20, 22, and 24 h). Interestingly, the statistical analysis showed a significant (*p*  < 0.0001) effect of the CBD on *S. oralis* growth at all tested times and with all included CBD concentrations above 3.12 μg/mL.

To evaluate the specific efficacy of each chosen CBD concentration relative to the control over the entire 24‐h period, a focused analysis was conducted. Derived from the overarching two‐way ANOVA model, this approach directly compared each treatment group with DMSO at all time points, allowing us to pinpoint significant differences across growth stages. As shown in Figure 3, the 6.25 and 12.50 µg/mL displayed nearly identical kinetic profiles to each other and, importantly, showed statistically significant suppression compared to the DMSO at all tested timepoints, confirming a stable and consistent antimicrobial effect throughout the 24 h. In contrast, the behavior of the 3.12 µg/mL concentration was distinct. While it initially showed significant inhibitory effects compared to the control, this effect was not sustained over time, starting at 16 h post‐exposure when *S. oralis* began to grow.

**Figure 4:**
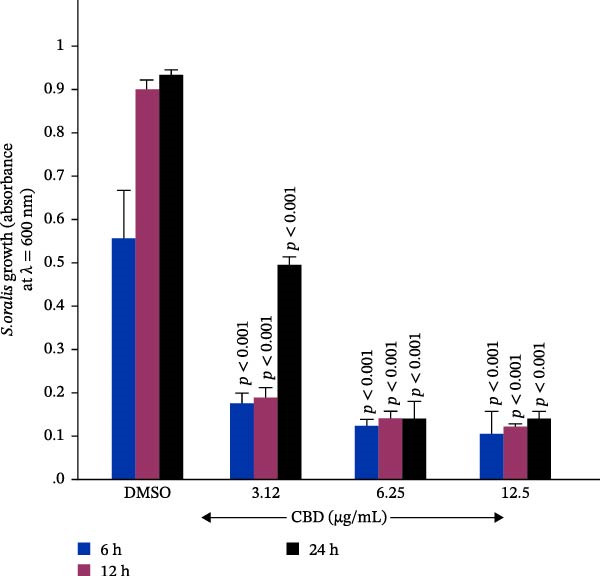
CBD inhibits *S. oralis* growth at early and late exposure periods. Selected exposure periods were used to monitor bacterial growth in the presence of CBD at various concentrations. Bacterial growth was followed by measuring absorbance at 600 nm. Four biological replicates were performed, and the means ± SD were presented. Statistical analysis was performed by comparing the test (presence of CBD) to the control (absence of CBD) using a one‐way ANOVA.

To confirm the inhibitory effect of CBD on bacterial growth, we selected specific exposure times (6, 12, and 24 h). We monitored *S. oralis* growth after exposure to selected CBD concentrations (3.12, 6.25, and 12.5 µg/mL). The obtained results were subjected to one‐way ANOVA analysis and are presented in Figure 4. It should be noted that even the low concentration (3.12 µg/mL) had significant bacterial growth inhibition at early timepoints (6 and 12 h) but lost this effect later (up to 24 h), while the other concentrations remained efficient in inhibiting the *S. oralis* growth. Furthermore, considering the effect of CBD on *S. oralis* growth (Figures 2–4), 6.25 µg/mL was determined as the MIC. With these results, we asked: What would the MBC of CBD be?

### 3.3. MBC

**Figure 5:**
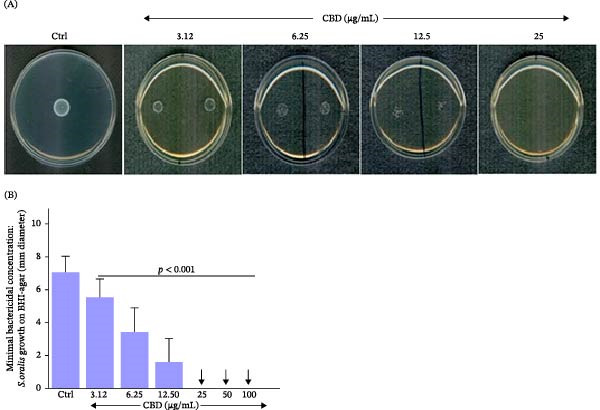
Minimal bactericidal concentration (MBC). *S. oralis* was pre‐exposed for 24 h to CBD, then subcultured by placing 5 µL of the cell suspension as a droplet onto the BHI‐Agar surface and incubated for 24 h at 37°C. The bacterial growth in the form of a halo was photographed (A), and the diameter of each halo was measured (B). Representative photos of 4 biological replicates were presented. Statistical significance was obtained when comparing the test (presence of CBD to the control (absence of CBD).

To determine the MBC, we subcultured the bacteria following 24 h of preexposure to CBD using BHI‐Agar plates. Figure 5A shows higher bacterial growth in the control (DMSO), while cells exposed to CBD had a lower growth rate. Indeed, at 3.12 µg/mL CBD, *S. oralis* showed a smaller bacterial halo than that of the control. It can also be noted that the bacterial growth halo decreases with increased concentrations of CBD. At 12.50 µg/mL, only discrete, isolated colonies are still observable, whereas at 25 µg/mL CBD, no bacterial growth halo was observed. Quantitative measurements of the halo diameter at each CBD concentration confirmed (Figure 5B) the decrease in *S. oralis* growth due to CBD. In this set of experiments, we demonstrated that 25 µg/mL CBD was the MBC for this product.

### 3.4. CBD Inhibits *S. oralis* Biofilm Formation

**Figure 6:**
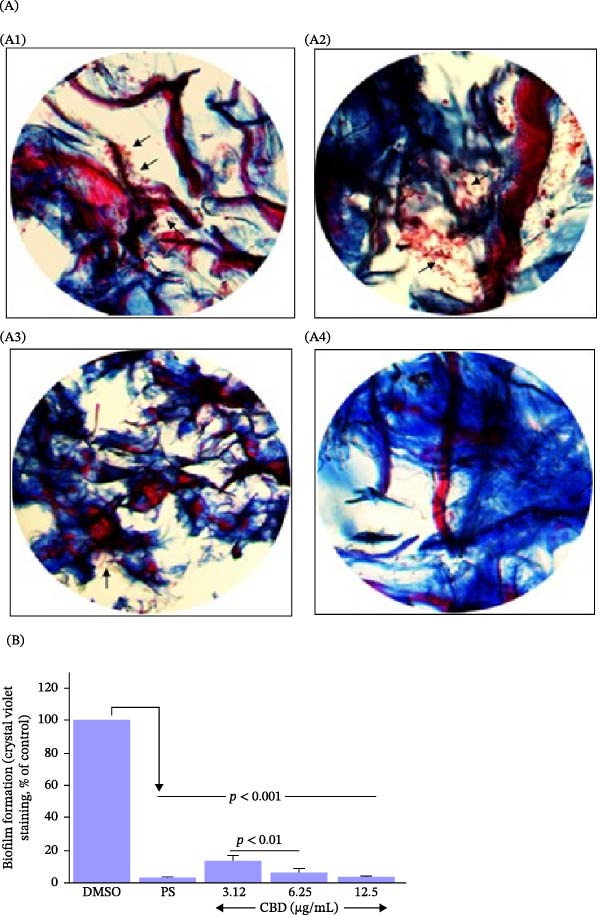
CBD inhibits biofilm formation by *S. oralis*. Bacterial cells were seeded into three‐dimensional collagen scaffolds and cultured for 3 days in the presence or absence (controls) of CBD at different concentrations. We refreshed the medium and the CBD treatment every 24 h. After 3‐day exposure to CBD, biofilm biomass of *S. oralis* was subjected to histological analysis (A) or crystal violet staining (B). Data were presented as mean ± SD of three biological replicates (*n* = 3). Only statistically significant comparisons are indicated in the graph. Statistically significant differences between groups were calculated using one‐way ANOVA. (A1) DMSO, (A2) 3.12 μg/mL, (A3) 6.25 μg/mL, and (A4) 12.5 μg/mL of CBD.

The effect of CBD on *S. oralis* biofilm formation was evaluated by histological and quantitative assays. Histological analysis (Figure 6A) revealed frequent red‐brown clumps of bacteria within the scaffold in the control group (arrows, Figure 6). Bacterial aggregates were also observable in the 3.12 μg/mL CBD group (arrows, Figure 6A, A2), but they were reduced in the 6.25 μg/mL group (arrows, Figure 6A) and markedly decreased in the 12.5 μg/mL group (Figure 6A, A4). To support these histological observations, biofilm biomass was quantified using CV staining. As shown in Figure 6B, varying concentrations of CBD led to a significant decrease in the *S. oralis* biofilm formation. Although biofilm formation was still observable at 3.12 (g/mL), in the 6.25 and 12.50 (g/mL) CBD concentrations, we observed a more pronounced reduction in biofilm formation. Interestingly, the effect of CBD on biofilm formation was not statistically different from that observed with the antibiotic (Figure 6B). Overall, histological findings corroborated the CV staining results, demonstrating the effectiveness of CBD in reducing *S. oralis* biofilm formation.

### 3.5. Gingival Epithelial Cells Exposed to CBD Showed Shapes and Adhesion Comparable to Those of the Control

**Figure 7:**
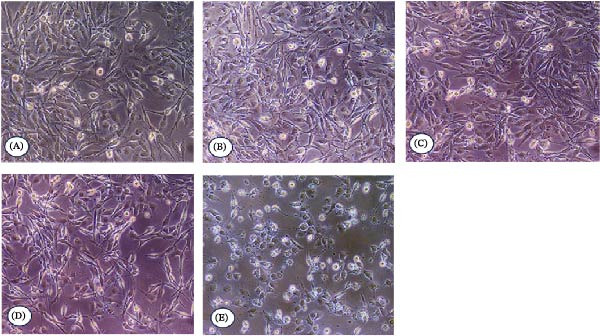
CBD does not affect gingival epithelial cell morphology and adhesion. Cells were seeded and immediately exposed to CBD and cultured for 24 h. Cell morphology and adhesion were observed under an inverted microscope and photographed. Representative photos out of four biological replicates (*n* = 4) were presented. (A) cells with culture medium, (B) cells with vehicle (DMSO), (C) cells with 3.12 µg/mL, (D) cells with 6.25 µg/mL, and (E) cells with 12.50 µg/mL of CBD.

Gingival epithelial cells were cultured for 24 h in the presence of CBD, and the cell morphology and adhesion were evaluated by optical microscopy. Cells exposed to vehicle (DMSO) (Figure 7B) exhibited high adhesion density, with an elongated morphology comparable to the control (cells cultured in medium without CBD or DMSO) (Figure 7A). Similarly, cells exposed to 3.12 µg/mL (Figure 7C) and 6.25 µg/mL (Figure 7D) of CBD showed morphologies and adhesion patterns comparable to those of the control groups (medium alone or with DMSO). Exposure to 12.50 µg/mL showed (Figure 7E) a slight difference in cell morphology as cells did not adopt the elongated shape observed in the control groups. However, based on our qualitative analysis, cell adhesion appeared to be maintained, with similar cell densities adhering to the culture plates (Figure 7E). These findings suggest that CBD concentrations up to 12.5 µg/mL did not markedly alter gingival epithelial cell adhesion or morphology.

### 3.6. CBD did not Reduce Human Gingival Epithelial Cell Viability/Proliferation

**Figure 8:**
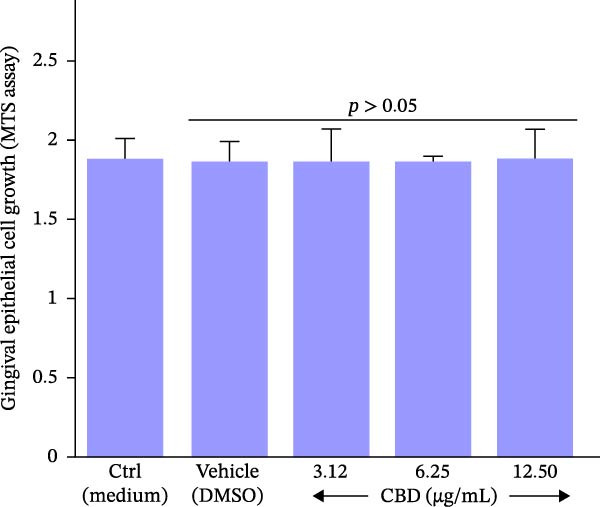
CBD maintaining human gingival epithelial cell viability. Cells were cultured for 24 h to adhere, then exposed to CBD at different concentrations for 24 h. The cell viability/proliferation was assessed using the MTS colorimetric assay. Cell viability was compared with the control group; no statistically significant differences were observed (ns: *p*  > 0.05), as determined by one‐way ANOVA (*n* = 5).

To further assess the compatibility of CBD with human gingival epithelial cells, cell viability/proliferation in CBD‐treated cells was evaluated using the MTS assay. As shown in Figure 8, no significant decrease in gingival epithelial cell viability was observed after 24 h of exposure to CBD. Interestingly, regardless of the CBD concentration (3.12, 6.25, or 12.50 µg/mL), cell viability was the same as that obtained with the control (medium alone) and the vehicle (DMSO). These results demonstrated that CBD concentrations up to 12.5 µg/mL did not reduce cell viability. Notably, this concentration range also showed inhibitory effects against *S. oralis* growth and biofilm formation.

## 4. Discussion

This in vitro study demonstrates, for the first time, that CBD affects *S. oralis* in two physiological states. CBD inhibited planktonic growth in a concentration‐dependent manner and reduced biofilm biomass, while histological analyses revealed a progressively disrupted biofilm architecture following CBD exposure. Given that oral antimicrobials must be effective against biofilm‐associated bacteria while maintaining compatibility with host tissues, we also evaluated gingival epithelial cell responses at the same concentration range. Although the antimicrobial and anti‐inflammatory potential of CBD in oral infections has recently been reviewed [12–14], the present findings add a specific experimental contribution by focusing on *S. oralis*, an early colonizer involved in the initial organization of oral biofilms. This distinction is important because most available studies have focused on overtly pathogenic species or mature disease‐associated biofilms.

In contrast, modulation of early colonizers may represent a preventive strategy for controlling biofilm development. [14, 15, 28]. Therefore, investigating the effects of CBD on *S. oralis*, a key early colonizer of the dental biofilm, is highly relevant for understanding its potential to modulate biofilm development and contribute to the control of biofilm‐associated diseases.

Natural plant‐derived compounds have attracted attention as alternative antibacterial agents, with CBD, the main non‐psychoactive cannabinoid of *Cannabis sativa*, standing out for its diverse biological activities [29, 30]. Recent studies have demonstrated its antimicrobial potential against Gram‐positive bacteria, including highly resistant species such as *Staphylococcus aureus*, *Streptococcus pneumoniae*, and *Clostridioides difficile* [14, 29, 31].

In the oral environment, biofilms pose a clinical challenge due to their intrinsic resistance to antimicrobial agents, underscoring the need to investigate new compounds such as CBD to control bacterial colonization and biofilm formation. The antimicrobial effect of CBD appears to involve damage to the bacterial membrane, inhibition of protein, DNA, RNA, and peptidoglycan synthesis, as well as disruption of the plasma membrane of Gram‐positive bacteria, inducing membrane hyperpolarization and impairing ion channel function [15, 30].

In our study, MIC and MBC analyses revealed that CBD is effective at relatively low concentrations against *S. oralis*, with an MIC of 6.25 µg/mL (Figures 2–4) and bactericidal activity observed at 25 µg/mL (Figure 5), demonstrating antimicrobial activity in the planktonic state. These findings are consistent with the broader literature, which shows that CBD can exert antimicrobial effects against various bacterial species. However, the concentrations required for inhibition vary considerably depending on the microorganism and experimental conditions. Blaskovich et al. [30] reported lower MIC values for several Gram‐positive bacteria (1–4 µg/mL) and selected Gram‐negative species (0.25–2 µg/mL), suggesting that *S. oralis* may be comparatively less susceptible to CBD under the conditions used in the present study. A systematic review focused on *Streptococcus* and *Staphylococcus* reported that CBD MICs span a wide range depending on the organism and study design, including values up to 50 µg/mL for *Streptococcus pyogenes*, suggesting that susceptibility may vary substantially across streptococcal species and experimental settings [31]. In the oral context, MIC/MBC values in the µg/mL range have also been reported for other streptococci. Recent studies have shown that CBD exhibits antibacterial and antibiofilm activity against oral bacteria, including cannabinoid‐sensitive streptococcal species and periodontopathogens. However, effective concentrations vary according to bacterial species, strain, biofilm model, and assay conditions [32–34].

Growth kinetics analysis further showed a concentration‐ and time‐dependent effect: while 3.12 µg/mL did not sustain complete inhibition after 24 h, it delayed bacterial proliferation by ~16 h. This temporal inhibition suggests that sub‐MIC concentrations of CBD may be biologically relevant in the context of oral biofilm development. Sub‐MIC levels of CBD in *S. mutans* delay growth phases, whereas MIC‐level concentrations sustain growth inhibition without bacterial recovery [14]. A similar time‐dependent pharmacodynamic profile has been reported for CBD against gram‐positive bacteria in CFU‐based time‐kill assays. Blaskovich et al. [30] showed that CBD exhibited rapid antibacterial activity against *S. aureus* within 3 h, with an MBC of 2 µg/mL, and time‐kill curves indicated concentration‐dependent bacterial killing. Importantly, regrowth after initial suppression has also been described in cannabinoid time‐kill settings, including re‐initiation of growth after 8 h, attributed to possible degradation or oxidation of CBD [35, 36], which supports the interpretation that delayed outgrowth at sub‐MIC can occur even when early inhibition is evident. Together, these results indicate that CBD interferes with bacterial growth dynamics, particularly at lower concentrations, rather than acting solely as an immediate bactericidal agent.

The antibiofilm activity of CBD against *S. oralis* was demonstrated through both quantitative and structural analyses. CV staining showed a significant reduction in biofilm biomass at all tested CBD concentrations (3.12–12.50 µg/mL), with more pronounced effects at higher concentrations, comparable to those of the control. These results suggest that CBD can be used at concentrations below 12.5 µg/mL to control *S. oralis* growth for up to 24 h efficiently. The CV assay is widely used for quantitative biofilm assessment, as illustrated by Wieczerza et al. [37], who used this method to evaluate the effects of cannabinoids on biofilms formed by bacteria associated with endodontic infections. Because CV staining primarily quantifies total biofilm biomass (cells and secreted extracellular matrices) rather than directly assessing bacterial viability, combining this approach with structural imaging strengthens data interpretation. This rationale is supported by previous studies investigating CBD activity against biofilms, in which CV–based minimum biofilm eradication concentration (MBEC)/minimum biofilm inhibitory concentration (MBIC) thresholds (≥70% inhibition relative to the growth control) are used as quantitative proxies for biofilm burden [29].

Histological analyses of biofilm sections formed on collagen membranes corroborated these findings, revealing a disrupted biofilm architecture and minimal residual bacterial clusters at the highest CBD concentrations. These observations are consistent with those reported by Barak et al. [14], who showed that CBD interferes with *S. mutans* biofilm formation, resulting in sparse, poorly organized biofilm structures, as visualized by confocal microscopy. Together, these convergent qualitative patterns support the antibiofilm activity of CBD against oral streptococci and reinforce that CBD compromises biofilm integrity beyond simple biomass reduction. This biomass and structure approach is consistent with prior CBD antibiofilm evidence in Gram‐positive biofilms: CBD was active against established *S. aureus* biofilms with MBEC ranging from 1 to 2 µg/mL for *Methicillin-Susceptible Staphylococcus aureus* (MSSA) and from 2 to 4 µg/mL for methicillin‐resistant *Staphylococcus aureus* (MRSA), and confocal microscopy demonstrated CBD penetration and killing within the biofilm [30]. It has also been reported that higher CBD concentrations were required to achieve >90% killing (32 µg/mL) than those suggested by CV MBEC values, emphasizing that biomass‐based assays and viability‐oriented imaging can yield different “effective concentrations” due to differences in assay conditions and readouts [30]. Mechanistically, this is compatible with the broader view that CBD’s lipophilic structure enables membrane interaction, promoting membrane disruption and increased permeability, which can plausibly impact both planktonic cells and biofilm organization [4]. In streptococcal biofilms specifically, independent antibiofilm literature supports the concept that biofilm architecture and matrix act as barriers to antimicrobial activity and that disrupting matrix components can markedly increase susceptibility, reinforcing why architectural disruption is a meaningful endpoint beyond biomass reduction [38].

From a translational perspective, the incorporation of CBD into oral formulations has been proposed for mouthrinses, gels, lozenges, and local delivery systems [15]. The study by Torabi et al. [39] demonstrated that the use of oral lozenges containing CBD resulted in a significant reduction in the abundance of *S. mutans* in saliva, suggesting that the effects observed in experimental models may translate into benefits within the complex oral environment. Although the specific mechanisms against *S. oralis* were not fully elucidated here, the literature suggests that CBD acts by disrupting the cell membrane, reducing extracellular polysaccharides [14], and inducing cell envelope stress [30]. This combination of actions explains the double effect observed: the inhibition of growth and the structural disruption of the biofilm, positioning CBD as a modulator of biofilm development rather than a classic bactericidal agent. Notably, cannabinoid‐infused mouthwashes containing CBD or CBG have demonstrated bactericidal efficacy comparable to 0.2% chlorhexidine, the gold standard mouthwash in dentistry, but without the considerable side effects commonly associated with chlorhexidine use [10, 11, 31]. These findings support the potential of cannabinoids as promising alternatives for oral care applications.

Furthermore, synergistic effects have been reported between CBD and other antimicrobial agents. Avraham et al. [40] demonstrated that the combination of triclosan and CBD exerted stronger antibacterial and antibiofilm effects than either compound alone. Both agents were shown to induce membrane hyperpolarization and reduce bacterial viability and adhesion. Such combinatorial approaches may be particularly valuable for preventing dental caries and controlling oral inflammation.

The effects of CBD on human gingival epithelial cells were also evaluated for adhesion, morphology, and viability. CBD concentrations up to 12.50 µg/mL were selected for these assays because they corresponded to the concentration range evaluated in the biofilm formation assays, which may be more relevant to the oral environment than the bactericidal concentration determined in the planktonic model. It was observed that at ≤12.50 µg/mL, CBD did not significantly reduce cell adhesion, alter cell morphology, or decrease cell viability after 24 h of exposure. These findings are partially consistent with a recent study by Bahraminia et al. [18], which investigated the effects of THC and CBD on gingival and skin keratinocytes and found that lower CBD concentrations did not impair cell metabolism, whereas concentrations ≥10 µg/mL were associated with reduced cell viability. In contrast, lower concentrations did not impair the cell metabolism. Since the concentration range evaluated in the present study overlaps with this threshold, the apparent difference between studies should be interpreted cautiously. Such differences may be related to the cell type, exposure conditions, assay methodology, and the concentration range evaluated.

Although the present study demonstrated that CBD inhibited *S. oralis* growth and biofilm formation under controlled laboratory conditions, these findings should be interpreted within the limitations of an in vitro model. The experiments in this study used a single‐species biofilm model with a single *S. oralis* strain, which does not fully reflect the strain‐dependent variability in antimicrobial susceptibility. In addition, this model does not reproduce the complexity of the oral cavity, including saliva, multispecies biofilms, host immune responses, tissue interactions, and systemic patient‐related factors. Therefore, further investigations should evaluate the effects of CBD in multispecies biofilm models and in additional oral bacterial species. Moreover, the cellular assays were performed in a single gingival epithelial cell line under in vitro conditions, which may not fully reproduce an in vivo microenvironment. Future ex vivo, in vivo, and clinical studies are necessary to confirm the translational potential of CBD as an antimicrobial agent in oral biofilm management. Despite these limitations, the present findings provide a relevant preliminary foundation for further investigations into the antimicrobial and antibiofilm properties of CBD in oral health contexts.

## 5. Conclusion

This study demonstrated the effectiveness of CBD at different concentrations in inhibiting the growth of *S. oralis* at both early and late exposure periods. CBD also had a significant effect on biofilm formation by *S. oralis*. Low and medium concentrations of CBD, which have antimicrobial effects, did not reduce the viability of human gingival epithelial cells. However, these findings should be interpreted with the limitations of an in vitro model in mind. Overall, this study provides preliminary evidence supporting further investigation of CBD as a potential antimicrobial molecule against *S. oralis*. Further studies are required to determine its safety, optimal concentration, delivery method, and clinical relevance for oral health applications.

## Author Contributions

**Celina Cruz Mainardes:** conceptualization, methodology, validation, formal analysis, investigation, data curation, writing – original draft, writing – review & editing. **Omayma Amri:** methodology, validation, investigation, data curation. **Denise M. Palomari Spolidorio:** writing – review & editing, supervision, project administration. **Mahmoud Rouabhia:** conceptualization, methodology, validation, formal analysis, investigation, data curation, funding acquisition, writing – original draft, writing – review & editing, supervision, project administration.

## Funding

This research was funded by the Natural Sciences and Engineering Research Council of Canada (NSERC) through the NSERC‐Discovery grant to MR (RGPIN‐2019‐04475). Celina Cruz Mainardes benefited from a scholarship from the São Paulo Research Foundation (FAPESP), Grant #2025/04500‐5. NSERC and FAPESP had no role in the study design, data collection and analysis, manuscript preparation, or the decision to publish.

## Disclosure

No formal study protocol was registered or publicly available. All authors have read and approved the final version of the manuscript. The authors had full access to all data in this study and took full responsibility for the integrity and accuracy of the data analysis.

## Conflicts of Interest

The authors declare no conflicts of interest.

## Data Availability

The authors confirm that the data supporting the findings of this study are available within the article.
